# Supplementary material for: Development of Novel Monoclonal Antibodies to Wheat Alpha-Amylases Associated with Grain Quality Problems That Are Increasing with Climate Change
Source: Plants (Basel). 2023 Nov 8;12(22):3798. doi: 10.3390/plants12223798 (PMC10675223; doi:10.3390/plants12223798)
Supplement: Supplementary file 1 [file plants-12-03798-s001.zip › Hauvermale_Supplemental Antibody Figure S1.pptx]

## Slide 1
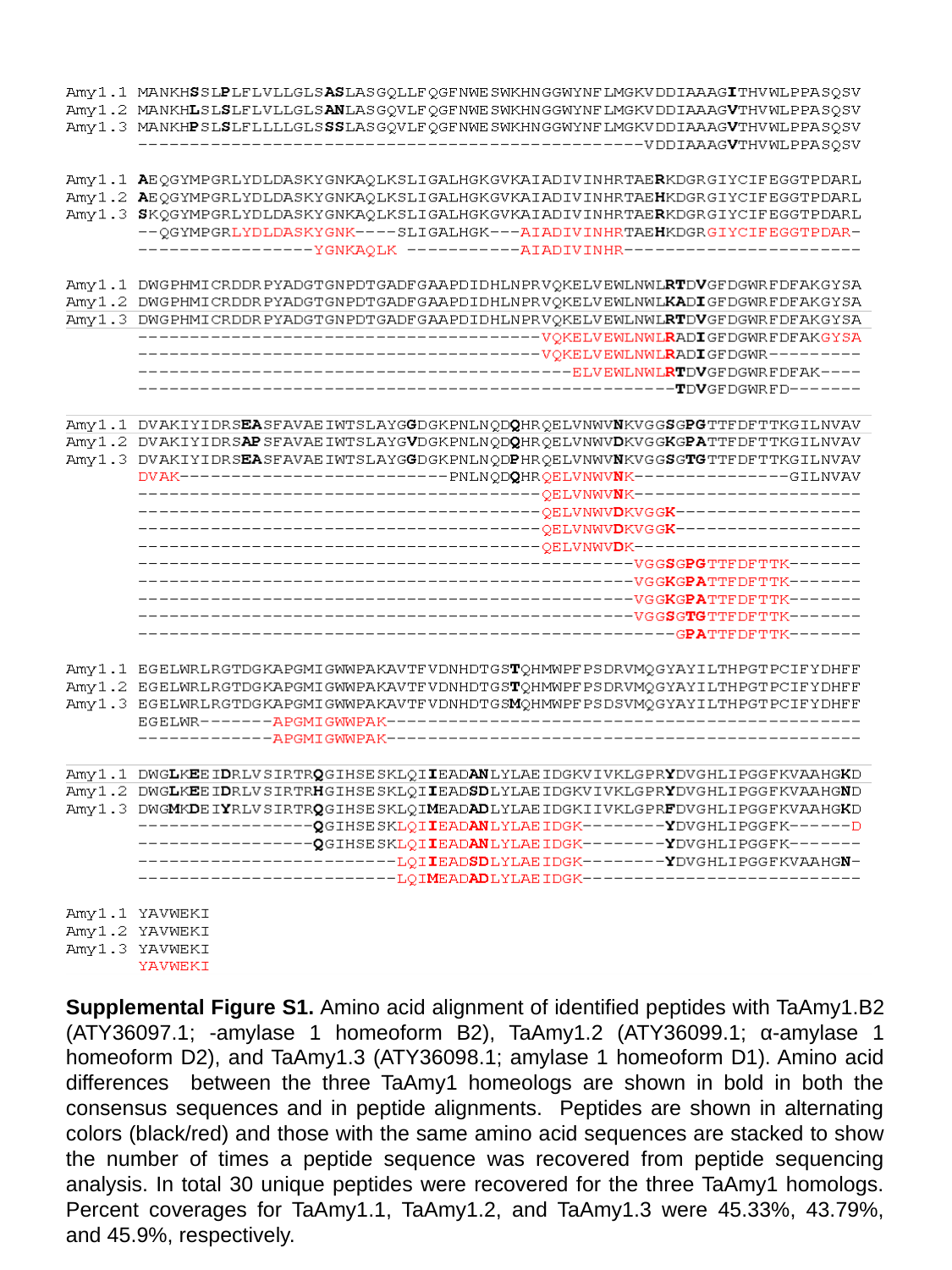

Supplemental Figure S1. Amino acid alignment of identified peptides with TaAmy1.B2 (ATY36097.1; -amylase 1 homeoform B2), TaAmy1.2 (ATY36099.1; α-amylase 1 homeoform D2), and TaAmy1.3 (ATY36098.1; amylase 1 homeoform D1). Amino acid differences between the three TaAmy1 homeologs are shown in bold in both the consensus sequences and in peptide alignments. Peptides are shown in alternating colors (black/red) and those with the same amino acid sequences are stacked to show the number of times a peptide sequence was recovered from peptide sequencing analysis. In total 30 unique peptides were recovered for the three TaAmy1 homologs. Percent coverages for TaAmy1.1, TaAmy1.2, and TaAmy1.3 were 45.33%, 43.79%, and 45.9%, respectively.
