# Supplementary material for: Development of Novel Monoclonal Antibodies to Wheat Alpha-Amylases Associated with Grain Quality Problems That Are Increasing with Climate Change
Source: Plants (Basel). 2023 Nov 8;12(22):3798. doi: 10.3390/plants12223798 (PMC10675223; doi:10.3390/plants12223798)
Supplement: Supplementary file 1 [file plants-12-03798-s001.zip › Hauvermale_Supplemental Antibody Figure S2.pptx]

## Slide 1
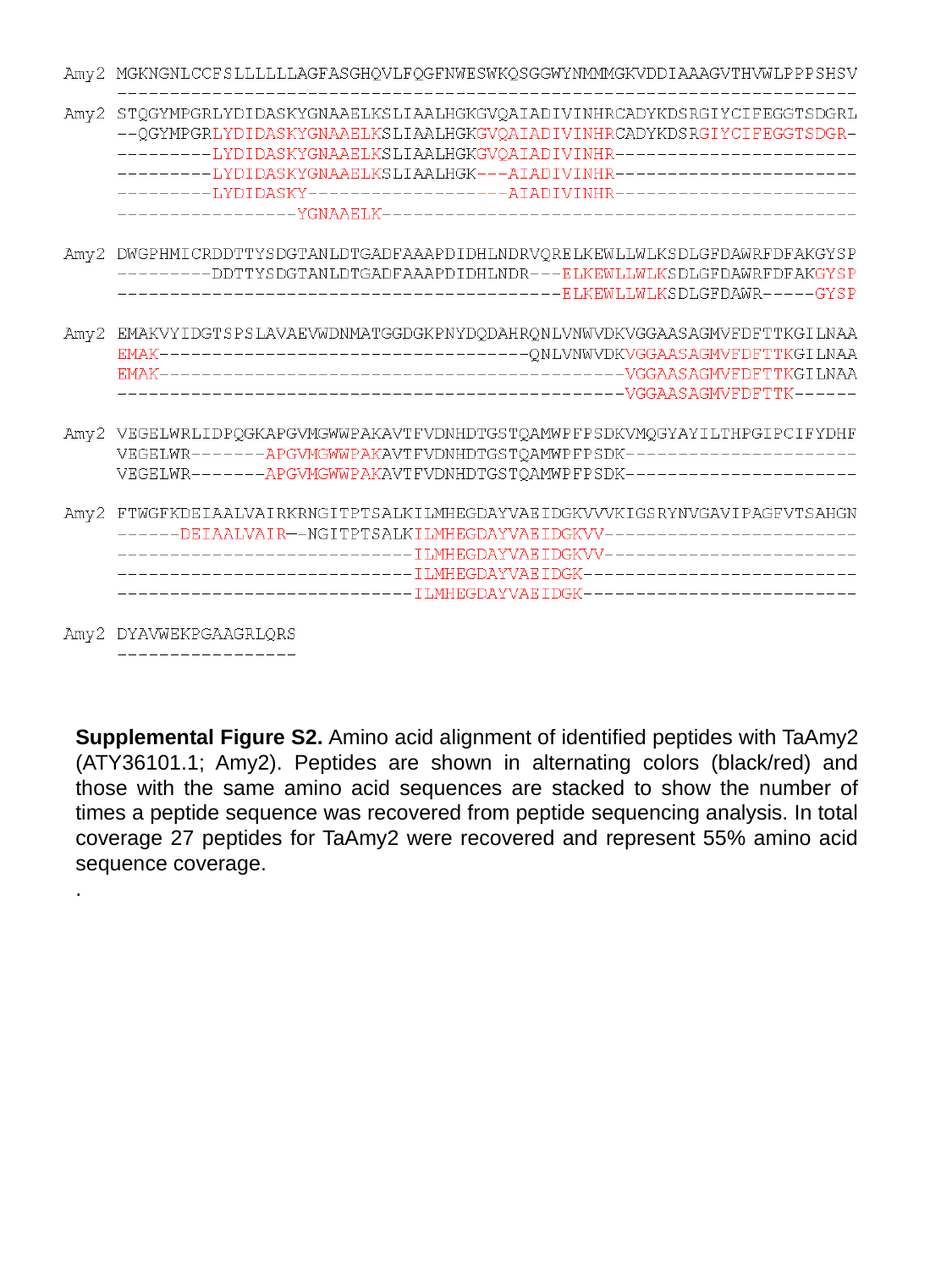

Supplemental Figure S2. Amino acid alignment of identified peptides with TaAmy2 (ATY36101.1; Amy2). Peptides are shown in alternating colors (black/red) and those with the same amino acid sequences are stacked to show the number of times a peptide sequence was recovered from peptide sequencing analysis. In total coverage 27 peptides for TaAmy2 were recovered and represent 55% amino acid sequence coverage.
.
