# Supplementary material for: Development of Novel Monoclonal Antibodies to Wheat Alpha-Amylases Associated with Grain Quality Problems That Are Increasing with Climate Change
Source: Plants (Basel). 2023 Nov 8;12(22):3798. doi: 10.3390/plants12223798 (PMC10675223; doi:10.3390/plants12223798)
Supplement: Supplementary file 1 [file plants-12-03798-s001.zip › Hauvermale_Supplemental Antibody Figure S3.pptx]

## Slide 1
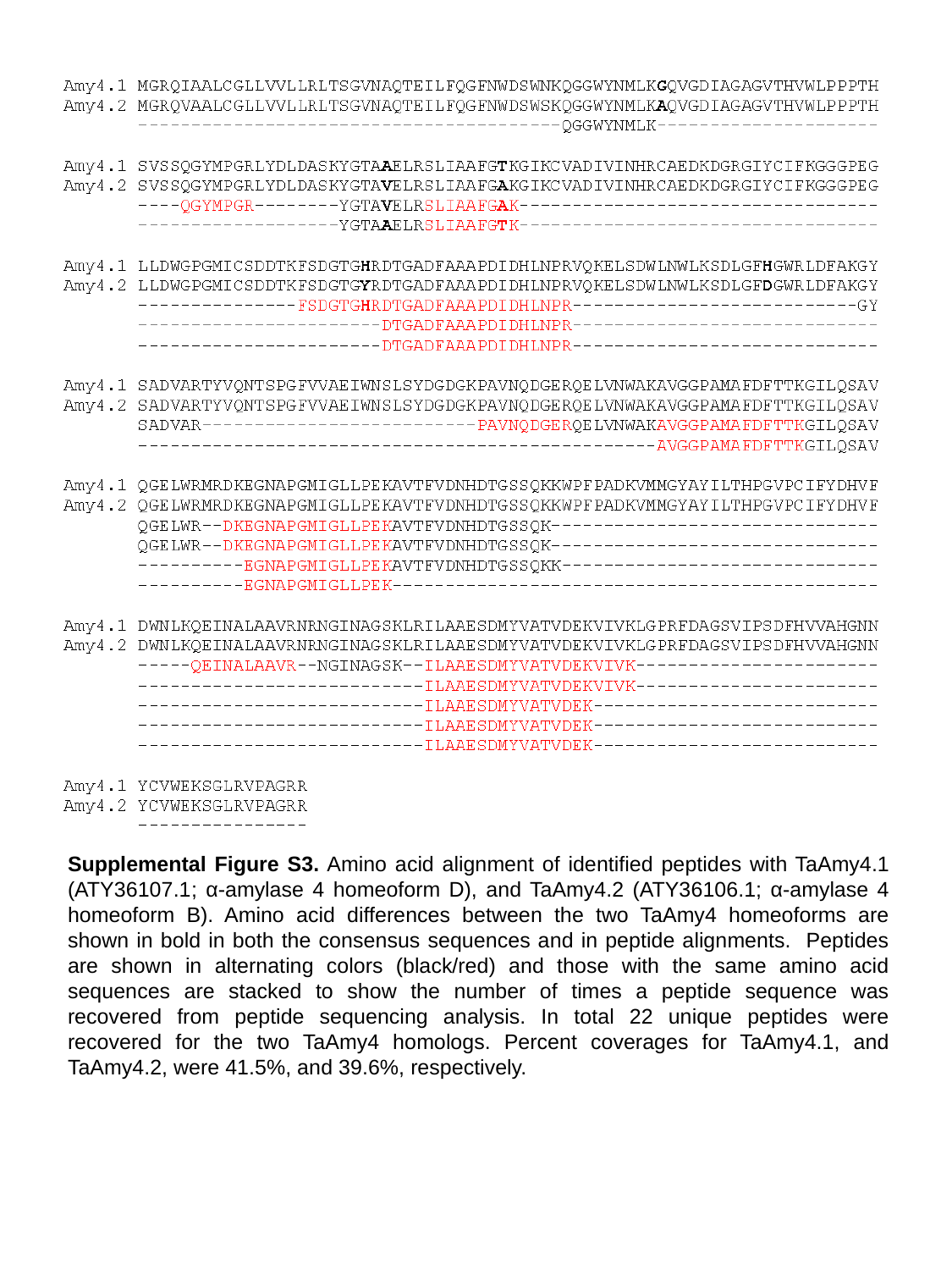

Supplemental Figure S3. Amino acid alignment of identified peptides with TaAmy4.1 (ATY36107.1; α-amylase 4 homeoform D), and TaAmy4.2 (ATY36106.1; α-amylase 4 homeoform B). Amino acid differences between the two TaAmy4 homeoforms are shown in bold in both the consensus sequences and in peptide alignments. Peptides are shown in alternating colors (black/red) and those with the same amino acid sequences are stacked to show the number of times a peptide sequence was recovered from peptide sequencing analysis. In total 22 unique peptides were recovered for the two TaAmy4 homologs. Percent coverages for TaAmy4.1, and TaAmy4.2, were 41.5%, and 39.6%, respectively.
