# Supplementary material for: Development of Novel Monoclonal Antibodies to Wheat Alpha-Amylases Associated with Grain Quality Problems That Are Increasing with Climate Change
Source: Plants (Basel). 2023 Nov 8;12(22):3798. doi: 10.3390/plants12223798 (PMC10675223; doi:10.3390/plants12223798)
Supplement: Supplementary file 1 [file plants-12-03798-s001.zip › Hauvermale_Supplemental Antibody Figure S5.pptx]

## Slide 1
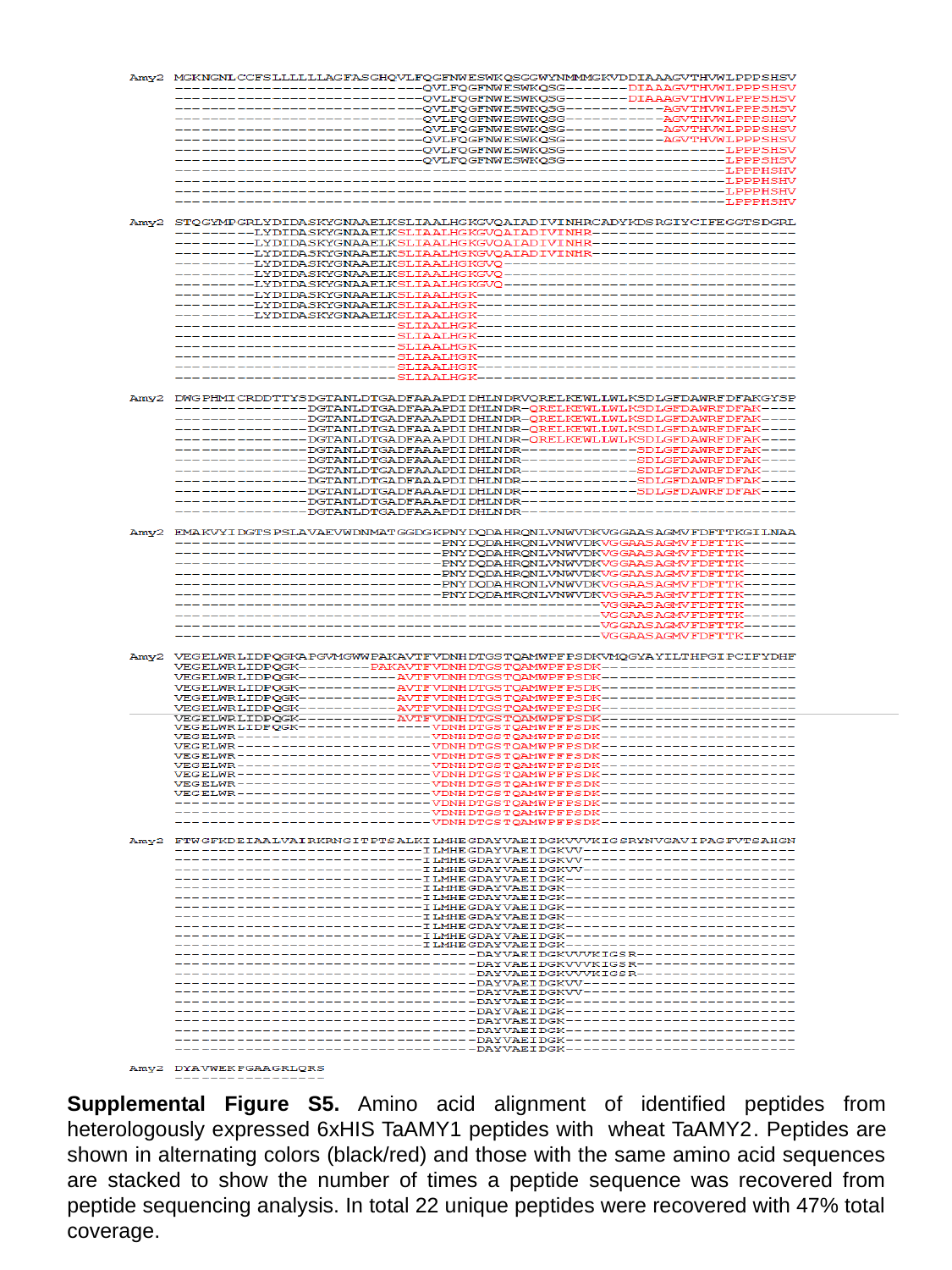

Supplemental Figure S5. Amino acid alignment of identified peptides from heterologously expressed 6xHIS TaAMY1 peptides with wheat TaAMY2. Peptides are shown in alternating colors (black/red) and those with the same amino acid sequences are stacked to show the number of times a peptide sequence was recovered from peptide sequencing analysis. In total 22 unique peptides were recovered with 47% total coverage.
