# Supplementary material for: Development of Novel Monoclonal Antibodies to Wheat Alpha-Amylases Associated with Grain Quality Problems That Are Increasing with Climate Change
Source: Plants (Basel). 2023 Nov 8;12(22):3798. doi: 10.3390/plants12223798 (PMC10675223; doi:10.3390/plants12223798)
Supplement: Supplementary file 1 [file plants-12-03798-s001.zip › Supplemental Table S1.pdf]

Supplemental Table S1: Primers used for cloning and RT-qPCR experiments.

Primer Name	Sequence 5'-3'	Reference*
Amy1_F_Court3	ATGGCGAACAAACACTCGTCC	
Amy1_R_Court3	TCATATTTTCTCCCATACGGCA	
Amy2_F_Court3	ATGGGGAGGAACGGCAAC	
Amy2_R_Court3	TCAGCTCCGTTGCAGTGTC	
Amy4_F_Court3	ATGGGGAGGCAGATCG	
Amy4_R_Court3	CTAGCGCCGCCCGGC	
F_Amy1_ResBgl	GCAGATCTCGCCCTTATGG	
R_Amy1_ResEco	GCCAGTGTGCTGGAATTC	
M13F	GTAAAACGACGGCCAG	[60]
M13T	CAGGAAACAGCTATGAC	[60]
T7F	TAATACGACTCACTATAGGG	[61]
T7R	CTAGTTATTGCTCAGCGGT	[61]
FAmy1Int_t7term	GCAAGGTCATCGTCAAGC	
RAmy1Int_t7prom	TCCACCTTGCCCATCAG	
Amy1F_Court4	GGCTCTACGACCTGGA	
Amy1R_Court5	GGGTGCGTGAGGATG	
Amy1F_CourtINT	GAAGCAAGAGATCAACGC	
Amy1R_CourtINT	GCCCATCAGGAAGTTGTAC	
Amy2F_CourtINT	TGAAGATCGGGTCCAGGTAC	
Amy2R_CourtINT	GTGGTTGCCGTTCCTCC	
Amy2F_CourtINT2	CGATCAGCCGCCACAGC	
Amy2R_CourtINT2	GCTGCGCCGACTACAAGGAC	
Amy4F_CourtINT	CGGAGAGCGACATGTACG	
Amy4R_CourtINT	CTTGAGCATGTTGTACCAGCC	
Amy4F_Court4	GCTCTACGACCTGGACG	
Amy4R_Court4	CGTAGCCCATCATGACCTTG	
RT-qPCR TaACT-F	TCAGCCGAGCGGGAAATTGT	[9]
RT-qPCR TaACT-R	CCTCTCTGCGCCAATCGT	[9]
RT-qPCR TaAMY1-F	TTCAACTGGGAGTCGTGGAA	[9]
RT-qPCR TaAMY1-R	CACCTTGCCCATCAGGAAGT	[9]
RT-qPCR TaAMY2-F	GGCCACCAAGTCCTCTTTCA	[9]
RT-qPCR TaAMY2-R	CACCTTGCCCGTCATCATGT	[9]
RT-qPCR TaAMY3-F	TTCTTTTCCAGGGGTTTAATTGGG	[9]
RT-qPCR TaAMY3-R	CTCCACCTTCCCTTGCATGA	[9]
RT-qPCR TaAMY3-GBF	ATCGTGGAAGACACAAGG	
RT-qPCR TaAMY3-GBR	CTTGGAGTTGAGGTTGTAGAG	
RT-qPCR TaAMY4-F	GAGGATGCGGGACAAGGAG	[9]
RT-qPCR TaAMY4-R	AACGGCCATTTCTTCTGCGA	[9]

* Unreferenced primers listed are novel.
